# The clinical efficacy of herbal medicines containing leeches in the treatment of coronary heart disease: a systematic review and meta-analysis

**DOI:** 10.3389/fphar.2025.1643611

**Published:** 2025-10-17

**Authors:** Zhao Ziyuan, Ye Di, Liang Mei, Jin Yujing, Zheng Jinghui

**Affiliations:** ^1^ Graduate School, Guangxi University of Chinese Medicine, Nanning, Guangxi, China; ^2^ Ruikang Hospital affiliated to Guangxi University of Chinese Medicine, Nanning, Guangxi, China

**Keywords:** coronary heart disease, leech, Chinese herbal medicine, meta-analysis, systematic review

## Abstract

**Background:**

Coronary heart disease (CHD) is a leading global cause of mortality, contributing to angina, arrhythmia, myocardial infarction, heart failure, and sudden death. Traditional treatments, including antiplatelet drugs, statins, β-blockers, and coronary stents, have notable side effects such as gastrointestinal bleeding and liver or muscle dysfunction, with post-operative stent complications. Recent studies have identified bioactive substances in leeches, particularly the natural anticoagulant hirudin, which inhibits thrombin and may help mitigate complications of coronary artery disease. Hirudin inhibits thrombin, reduces platelet aggregation, and lowers thrombosis risk. This meta-analysis evaluates the clinical efficacy and safety of Chinese herbal medicines containing leech in treating CHD, focusing on cardiovascular outcomes and adverse events.

**Methods:**

A systematic search of databases including PubMed, EMBASE, Web of Science, Cochrane Library, China National Knowledge Infrastructure (CNKI), WanFang Data (Wanfang), and VIP China Science and Technology Journal Database (VIP) was conducted to identify randomized controlled trials (RCTs) involving leech-containing Chinese herbal medicines for CHD patients up until February 2025. Key efficacy outcomes analyzed were total effective rate, ECG efficacy, and hemorheological parameters, while adverse event rates assessed treatment safety. The meta-analysis used Standardized Mean Difference (SMD) and 95% confidence intervals (CI) to assess efficacy, and Odds Ratio (OR) with 95% CI for safety. Subgroup analyses examined the relationship between therapeutic effects and patient characteristics.

**Results:**

Sixty-five studies involving 7,221 patients were included. The results showed that leech-based treatments significantly improved the total effective rate [OR = 3.70, 95% CI (3.19, 4.31), Z = 17.05, P < 0.00001] and ECG efficacy [OR = 2.58, 95% CI (2.23, 2.99), P < 0.0001], along with significant improvements in hemorheological parameters. Subgroup analysis indicated leech treatments were particularly effective for improving the total effective rate, ECG outcomes, and hemorheological indices. Importantly, adverse event rates did not increase compared to conventional treatments.

**Conclusion:**

Chinese herbal medicines containing leeches provide significant clinical benefits for CHD, particularly in improving ECG outcomes and blood parameters. These findings suggest that leech-based treatments are both effective and safe, with no increase in adverse events.

**Systematic Review Registration:**

https://www.crd.york.ac.uk/prospero/, Identifier CRD42024564675.

## 1 Introduction

Coronary heart disease is primarily caused by atherosclerosis or functional abnormalities in the coronary arteries, leading to myocardial ischemia, hypoxia, and necrosis. The disease’s primary clinical manifestations include angina pectoris, arrhythmia, and myocardial infarction (Pothineni et al., 2017). As one of the leading causes of global mortality, CHD accounts for approximately 9.31 million deaths annually, or nearly half of all cardiovascular disease-related deaths (Katta et al., 2021). The management of CHD typically involves antiplatelet agents, lipid-lowering drugs, β-blockers, and coronary stent implantation. However, these treatments are not without limitations, as prolonged use of these drugs can lead to adverse effects such as gastrointestinal bleeding, muscle and liver dysfunction, and complications from stent implantation (Passacquale et al., 2022; Mao et al., 2021; Zhang et al., 2024; Xin et al., 2023; Shan-Shan and Lu, 2022).

In light of the potential drawbacks of conventional therapies, recent research has focused on alternative treatments derived from traditional Chinese medicine (TCM) (Chunmiao et al., 2024). Substances in leeches, particularly hirudin, have garnered attention for their anticoagulant, anti-inflammatory, and fibrinolytic properties (Wu et al., 2024; Junren et al., 2021; Lemke and Vilcinskas, 2020). Hirudin directly inhibits thrombin activity, reduces platelet aggregation, and improves vascular health. Many randomized controlled trials have examined the efficacy of Chinese patent medicines containing leech components for treating CHD. However, no comprehensive comparison of the efficacy of different leech-containing medicines have been conducted. This study aims to bridge this gap by conducting a meta-analysis of RCTs to compare the efficacy and safety of these treatments.

## 2 Materials and methods

This meta-analysis was conducted in accordance with the PRISMA guidelines of the Cochrane Handbook and was registered on the PROSPERO website. The registration number (CRD42024564675).

### 2.1 Search strategy

A systematic search was performed across multiple key medical databases, including PubMed, Embase, Cochrane Library, Web of Science, CNKI, Wanfang, and VIP, covering publications from their inception until February 2025. Keywords such as “Leeches,” “Coronary Disease,” “Angina,” “Heart Disease,” “Acute Coronary Syndromes,” and “Randomized Controlled Trial (RCT)” were used to identify relevant studies. In addition, experts in the field were consulted to gather unpublished data and ensure a comprehensive search. Detailed search formula table is attached as an appendix.

### 2.2 Study selection

The studies were screened according to the PICOS framework. Eligible studies included RCTs evaluating the efficacy of Chinese herbal medicines containing leech components in CHD patients. Studies were excluded if they did not clearly mention the use of leech components, had incomplete data, or lacked scientific rigor (Table 1).

**TABLE 1:** Inclusion and exclusion criteria table.

Parameter	Inclusion criteria
Population	Patients with a definite diagnosis of coronary heart disease (diagnostic criteria were in accordance with relevant domestic and foreign guidelines) were enrolled
Intervention	Chinese herbal medicines containing leech ingredients were used alone or in combination with conventional treatment
Comparator	Conventional treatment, placebo or other Chinese medicine;
Outcome	The improvement rate of angina symptoms, electrocardiogram improvement rate, hemorheology index, incidence of major cardiovascular events and adverse drug reactions were recorded.
Study design	Being an RCT in either parallel or cross-over design
Exclusion criteria	Non-RCT studies, those not clearly mentioning the components of leeches, incomplete data or inability to extract valid data, studies with a follow-up period shorter than 4 weeks or lacking scientific evidence, as well as low-quality studies that have been repeatedly published.

This meta-analysis aims to evaluate the efficacy and safety of traditional Chinese medicines containing leech in the treatment of CHD by including high-quality RCTs. The inclusion criteria focus on studies with clear diagnostic standards for CHD, encompassing patients of any age or sex with stable or unstable angina. The interventions include the use of leech-containing traditional Chinese medicine, either as monotherapy or combined with conventional western medicine, compared with control groups receiving standard treatments, placebo, or TCM without leech. Key outcomes are categorized into primary outcomes, such as angina relief rate and ECG improvement rate, and secondary outcomes, including hemorheology and cardiac function indexes, incidence of major cardiovascular events, and drug-related adverse reactions. To ensure reliability, included studies must provide a clear description of randomization, control settings, and intervention measures, along with complete baseline data, post-treatment results, and statistical analyses. This comprehensive approach aims to provide robust evidence for the role of leech-containing TCM in CHD management.

Studies were excluded from this analysis if they: (1) Non-rct studies (such as observational studies, case reports, reviews, etc.); (2) The leech component was not explicitly mentioned in the intervention; (3) Incomplete data or unable to extract valid data; (4) The duration of follow-up was less than 4 weeks or the intervention lacked scientific evidence; (5) Duplicate published studies, and only the versions with complete data or higher quality were included.

### 2.3 Data extraction and quality assessment

Two investigators independently completed data extraction and cross-checked the extracted data. Disagreements were resolved through consultation or the intervention of third-party experts. The study authors; Time of publication; The sample size; Gender of the patient; Mean age; Type of disease; Primary outcome measures. If the data units are different for each parameter, we convert them to the most commonly used units.

The Cochrane Bias risk assessment tool was used to evaluate the quality of the included studies from the following six dimensions: 1. Random sequence generation (selection bias); 2. Allocation concealment (selection bias); 3. Blinded implementation (implementation bias and detection bias); 4. Data integrity (loss of follow-up bias); Selective reporting (reporting bias); 5. 6. Other sources of bias. According to the scoring results, the quality of the studies was divided into “high risk of bias”, “low risk of bias” and “uncertain risk of bias”.

Meta-analysis was performed using RevMan 5.4 software provided by Cochrane Collaboration. Relative risk difference (RD) and Mean difference (MD) were used as effect measures, and the point estimate of each effect size and its 95% confidence interval (95%CI) were reported. The test of heterogeneity was conducted by χ2 test (test level α = 0.1), and the degree of heterogeneity was evaluated by I^2^ statistic. Considering the possible statistical heterogeneity between studies, a random-effects model was used to integrate the results of various studies more accurately. In addition, funnel plots were used to assess the possibility of publication bias during the analysis of effect measures.

## 3 Results

### 3.1 Results of literature search

**FIGURE 1:**
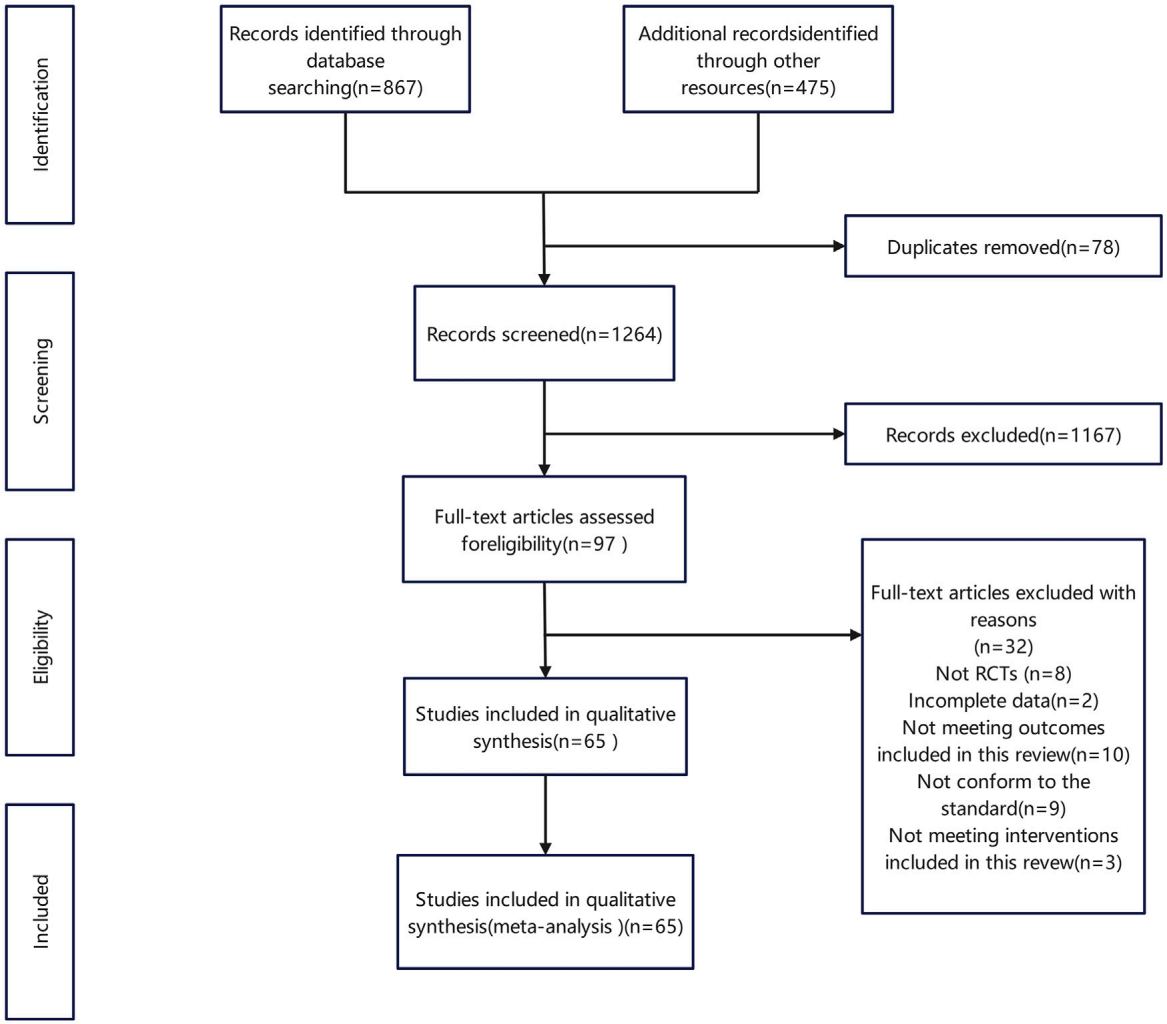
Flow chart of literature screening.

A total of 1,342 studies were initially retrieved, and after removing duplicates and screening titles and abstracts, 97 studies were shortlisted for full-text review as shown in Figure 1. Ultimately, 65 studies (Wu et al., 2001; Tong et al., 2001; Zhongtian and Rihui, 2001; Jian and Xudong, 2002; Jing et al., 2002; Taojin, 2002; Xianming et al., 2002; Yuxia et al., 2002; Zehong et al., 2002; Chunjian et al., 2003; Haijie and Changshi, 2003; Jinghe, 2003; Linfeng, 2003; Yanhong et al., 2003; Junjiang et al., 2004; Shaohua, 2004; Sujuan and Haijun, 2004; Lifang and Guofeng, 2005; Shougang, 2005; Xiaoyan and Bainian, 2006; Zhi-Min et al., 2006; Chunyan, 2007; Dongsheng and Jimin, 2008; Qingfan and Hezhong, 2008; Tangheng, 2008; Rongxing and Linlin, 2009; Shugang et al., 2009; Weidong and Lihua, 2009; Yunhai, 2009; Changming, 2010; Cuixia and Cunji, 2010; Haiyan, 2010; Shiyao, 2010; Xiaojun, 2010; Yong, 2010; Jun et al., 2011; Leilei, 2011; Quanyou, 2011; Shaofeng and Xinfeng, 2011; Xiao-Mei, 2011; Yongchao et al., 2011; Haiqing, 2012; Jinbao et al., 2012; Tingguo et al., 2012; Xinnian and Lu, 2012; Jinliang and Yunyi, 2013; Wei et al., 2013; Yu, 2013; Zongsheng, 2013; Cailiang et al., 2014; Sujuan et al., 2014; Yanbo, 2014; Youjun, 2014; Xuexu et al., 2014; Hongying et al., 2015; Zhaomei and Lian, 2015; Zhijing et al., 2016; Hongying et al., 2016; Jianmin et al., 2016; Mao and Qiang, 2017; Zhixue et al., 2017; Wenhui and Shiliang, 2019; Wenhua et al., 2020; Fei, 2021; Laiqiang, 2021), involving 7,221 patients, met the inclusion criteria.

### 3.2 Basic characteristics of the included literature

After systematic screening and evaluation, a total of 7,221 patients were enrolled in this study, including 3,795 patients in the experimental group and 3,426 patients in the control group. All studies were RCTS.

The studies included patients with stable or unstable angina, with a mean age of 50–70 years in most studies. Of the 65 studies, 31 primarily focused on coronary heart disease (Wu et al., 2001; Jian and Xudong, 2002; Taojin, 2002; Yuxia et al., 2002; Zehong et al., 2002; Linfeng, 2003; Sujuan and Haijun, 2004; Xiaoyan and Bainian, 2006; Zhi-Min et al., 2006; Chunyan, 2007; Dongsheng and Jimin, 2008; Tangheng, 2008; Weidong and Lihua, 2009; Yunhai, 2009; Changming, 2010; Haiyan, 2010; Shiyao, 2010; Jun et al., 2011; Leilei, 2011; Xiao-Mei, 2011; Yongchao et al., 2011; Haiqing, 2012; Tingguo et al., 2012; Xinnian and Lu, 2012; Jinliang and Yunyi, 2013; Wei et al., 2013; Sujuan et al., 2014; Yanbo, 2014; Youjun, 2014; Hongying et al., 2016; Jianmin et al., 2016), 23 on unstable angina (Tong et al., 2001; Zhongtian and Rihui, 2001; Jing et al., 2002; Chunjian et al., 2003; Haijie and Changshi, 2003; Yanhong et al., 2003; Junjiang et al., 2004; Shaohua, 2004; Lifang and Guofeng, 2005; Shougang, 2005; Qingfan and Hezhong, 2008; Rongxing and Linlin, 2009; Shugang et al., 2009; Cuixia and Cunji, 2010; Xiaojun, 2010; Yong, 2010; Quanyou, 2011; Jinbao et al., 2012; Yu, 2013; Zongsheng, 2013; Hongying et al., 2015; Mao and Qiang, 2017; Wenhua et al., 2020), 9 on stable angina pectoris (Xianming et al., 2002; Jinghe, 2003; Shaofeng and Xinfeng, 2011; Cailiang et al., 2014; Xuexu et al., 2014; Zhaomei and Lian, 2015; Wenhui and Shiliang, 2019; Fei, 2021; Laiqiang, 2021) and 2 on general angina symptoms (Zhijing et al., 2016; Zhixue et al., 2017). The detailed basic characteristics of the included studies are shown in Table 2.

**TABLE 2:** Evaluation table of literature quality.

Author	Year	Cases	Male/Female		Age (mean+SD)		Variety of disease	Outcome
			T	C				
Xue fei	2021	T:30 C:30	17/13	16/14	T:65.21±4.42	C:65.21±4.46	Stable angina pectoris	[1] [4] [5] [6]
Wang lai qiang	2021	T:50 C:50	27/23	25/25	T:63.47±5.89	C:62.56±6.57	Stable angina pectoris	[1] [2] [4] [7]
Liang wen hua	2020	T:49 C:53	57/45		T+C:56.15±9.24		Unstable angina	[1] [4] [5] [6] [7] [12]
Fan wen hui	2019	T:60 C:60	30/30	32/28	T:58.50±4.73	C:57.40 ±5.03	Stable angina pectoris	[1] [2] [4] [14] [15] [16] [17]
Ye mao	2017	T:60 C:60	34/26	33/27	T:61.7±15.5	C:61.5±15.3	Unstable angina	[1] [2] [12] [13] [14] [15] [16] [17] [5] [6]
Tian zhi xue	2017	T:40 C:40	28/12	27/13	T:60.3±5.4	C:60.8±5.7	Angina	[1] [4] [5] [6] [7] [8]
Yao hong ying	2016	T:30 C:30	17/13	15/15	T:52.78±2.56	C:53.69±3.23	Coronary heart disease	[1] [14] [15] [17]
Liin zhi jing	2016	T:150 C:150	79/71	76/74	T:65.2	C:64.1	Angina	[1] [12] [13] [20]
Dai jian min	2016	T:37 C:36	Not mentioned	Not mentioned	Not mentioned	Not mentioned	Coronary heart disease	[1] [2] [4] [14] [15] [16] [17]
Li hong ying	2015	T:52 C:54	31/21	33/21	Not mentioned	Not mentioned	Unstable angina	[1] [2] [4]
Deng zhao mei	2015	T:30 C:30	35/25		T+C:57.7±5.91		Stable angina pectoris	[1] [2]
Zhang you jun	2014	T:56 C:50	35/21	32/18	T:62	C:61	Coronary heart disease	[1] [2] [3]
Yang su juan	2014	T:96 C:104	44/52	49/55	Not mentioned	Not mentioned	Coronary heart disease	[1] [12] [13]
Su xue xu	2014	T:30 C:30	16/14	15/15	T:64.4±12.2	C:67.8±12.4	Stable angina pectoris	[1] [2]
Peng cai liang	2014	T:40 C:40	Not mentioned	Not mentioned	Not mentioned	Not mentioned	Stable angina pectoris	[1] [12] [13] [20]
Li yan bo	2014	T:50 C:50	28/22	24/26	T:64.5	C:66.5	Coronary heart disease	[1]
Wu jin liang	2013	T:156 C:98	90/66	58/40	T:70	C:69.5	Coronary heart disease	[1] [5] [6] [7] [8] [14] [15] [16]
Shang yu	2013	T:66 C:56	42/24	36/20	T:67±4.9	C:68±3.8	Unstable angina	[1] [5] [6] [10] [11] [7]
Huang wei	2013	T:72 C:36	40/32	20/16	T:57±7	C:57±8	Coronary heart disease	[1] [2]
Du zong sheng	2013	T:30 C:30	18/12	17/13	T:51.8±15.4	C:49.8 ±14.7	Unstable angina	[1] [12] [13] [20] [23]
Yu hai qing	2012	T:103 C:48	68/35	32/16	T+C:59.2		Coronary heart disease	[1] [2] [5] [6]
Shi jin bao	2012	T:35 C:32	20/15	18/14	T:58.1	C:60.2	Unstable angina	[1] [2] [4]
Lu ting guo	2012	T:80 C:80	45/35	43/37	T:48.3	C:47.5	Coronary heart disease	[1] [2] [12] [13]
Liu xin nian	2012	T:31 C:30	17/14	16/14	T:58.15±5.13	C:56.45±5.65	Coronary heart disease	[20] [21] [22] [23]
Zhu lei lei	2011	T:23 C:23	15/8	14/9	T:58 ±3.23	C:56 ±3.29	Coronary heart disease	[1] [2]
Zhang yong chao	2011	T:40 C:40	19/21	20/20	T:62.25±10.72	C:61.78±11.32	Coronary heart disease	[2] [12]
Ma xiao mei	2011	T:60 C:60	36/24	38/22	T:58	C:59	Coronary heart disease	[1] [2]
Li jun	2011	T:36 C:36	21/15	20/16	T:68±9	C:67±8	Coronary heart disease	[1] [24] [25]
Gong quan you	2011	T:70 C:70	42/28	40/30	Not mentioned	Not mentioned	Unstable angina	[1] [2]
Cai shao feng	2011	T:52 C:50	37/15	33/17	Not mentioned	Not mentioned	Stable angina pectoris	[1] [2]
Lu shi yao	2010	T:43 C:42	28/15	24/18	T:62.6	C:64.1	Coronary heart disease	[1]
Liu chang ming	2010	T:60 C:60	42/18	38/22	Not mentioned	Not mentioned	Coronary heart disease	[1]
Liu cui xia	2010	T:40 C:38	28/12	29/9	T:57±8	C:56±9	Unstable angina	[1]
Li xiao jun	2010	T:30 C:30	42/18		T+C:52±7		Unstable angina	[1]
Jin yong	2010	T:62 C:50	40/22	36/14	T:80	C:79	Unstable angina	[1] [2]
Huang hai yan	2010	T:42 C:40	22/20	21/19	T:58.2	C:56.2	Coronary heart disease	[1] [2]
Yuan shu gang	2009	T:60 C:60	38/22	40/20	T:67.15	C:68.12	Unstable angina	[1]
Ni wei dong	2009	T:36 C:32	42/26		T+C:52±7.5		Coronary heart disease	[1]
Chen rong xing	2009	T:60 C:58	40/20	39/19	Not mentioned	Not mentioned	Unstable angina	[2] [12] [13]
Cai yun hai	2009	T:69 C:66	35/34	31/35	T:46.39±10.17	C:45.12±8.97	Coronary heart disease	[1]
Zhou tang heng	2008	T:48 C:42	30/18	29/13	T:64	C:61	Coronary heart disease	[1] [2]
Zhang qing fan	2008	T:57 C:56	31/26	30/26	Not mentioned	Not mentioned	Unstable angina	[1] [2]
Chen dong sheng	2008	T:60 C:40	44/16	30/10	T:56±1.56	C:56±1.53	Coronary heart disease	[1] [2]
Yan chun yan	2007	T:60 C:60	81/39		Not mentioned	Not mentioned	Coronary heart disease	[1] [23]
Zhang zhi min	2006	T:250 C:250	163/87	166/84	T:55.6±20.4	C:54.3±17.7	Coronary heart disease	[14] [15] [16] [17]
Yang xiao yan	2006	T:40 C:40	24/16	23/17	T:55.7±4.3	C:54.9±4.9	Coronary heart disease	[1] [24] [25]
Jiao shou gang	2005	T:50 C:42	28/22	24/18	T:58.6	C:56.7	Unstable angina	[1] [2] [12] [14] [15] [17]
Chen li fang	2005	T:31 C:30	18/13	19/11	T:65.5	C:63.61	Unstable angina	[1] [2]
Zhang shao hua	2004	T:21 C:18	17/4	15/3	T:68.7	C:68.1	Unstable angina	[1] [20] [21] [22] [23]
Zhang jun jiang	2004	T:42 C:42	32/10	36/6	Not mentioned	Not mentioned	Unstable angina	[1] [6] [10]
Yang su juan	2004	T:120 C:80	86/34	58/22	Not mentioned	Not mentioned	Coronary heart disease	[1] [2]
Yu hai jie	2003	T:22 C:22	19/3	17/5	T:59.2	C:61.4	Unstable angina	[1] [2]
Wang yan hong	2003	T:60 C:40	41/19	28/12	T:68.3±6.4	C:67.4±6.9	Unstable angina	[1] [2] [5] [6] [8]
Shang jing he	2003	T:66 C:60	40/26	40/20	T:56.3	C:60.5	Stable angina pectoris	[1] [2] [5] [6] [7] [14] [15] [16]
Niu chun jian	2003	T:30 C:30	16/14	15/15	T:60.7	C:61.4	Unstable angina	[1] [12] [7] [14] [15] [10]
Liao lin feng	2003	T:40 C:25	27/13	14/11	T:62.43	C:61.56	Coronary heart disease	[1] [2] [5] [6] [7] [8]
Zhao ze hong	2002	T:175 C:121	195/101		T+C:63.2±9.7		Coronary heart disease	[1] [2] [12]
Zhao yu xia	2002	T:31 C:31	18/13	28/3	T:57±12	C:54±11	Coronary heart disease	[14] [15] [16]
Xue jing	2002	T:29 C:29	16/13	15/14	T:61.2	C:60.5	Unstable angina	[1] [2] [14] [15] [7] [19]
Li tao jin	2002	T:36 C:36	20/16	18/18	T:60.2±6.56	C:59.61±7.26	Coronary heart disease	[1] [2] [4] [18]
Fu jian	2002	T:45 C:40	26/19	24/16	T:61±7.2	C:59±6.2	Coronary heart disease	[1] [2] [3]
Fang xian ming	2002	T:30 C:30	23/7	24/6	T:56.8	C:57.2	Stable angina pectoris	[1] [2] [3]
Xu zhong tian	2001	T:52 C:52	38/14	34/18	Not mentioned	Not mentioned	Unstable angina	[1] [5] [6] [20] [9] [11]
Wang tong	2001	T:82 C:80	60/22	60/20	T+C:51.5±3.2		Unstable angina	[1] [2] [5] [6] [10] [9]
Wang ling yun	2001	T:100 C:100	107/93		T+C:56.3		Coronary heart disease	[1] [2]

T, experimental group, C, control group; A, Chinese patent medicine containing leech ingredients; B, conventional treatment; Efficacy evaluation indexes include; [1] Effective; [2] ECG efficacy; [3] Efficacy of main symptoms; [4] TCM symptom points; [5] Whole blood viscosity; [6] plasma viscosity (high + low); [7] Fibrinogen; [8] Hematocrit; [9] Sedimentation rate equation; [10] Platelet adhesion rate; [11] hematocrit; [12] frequency of angina attacks; [13] Duration of angina; [14] Total cholesterol; [15] Triglycerides; [16] High-density lipoprotein; [17] Low-density lipoprotein; [18] Blood glucose; [19] Prothrombin time; [20] ST-T changes; [21] Sum of ST changes; [22] systolic blood pressure; [23] heart rate; [24] Cardiac ejection fraction; [25] Left ventricular end-diastolic diameter.

### 3.3 Quality evaluation of included studies

**FIGURE 2:**
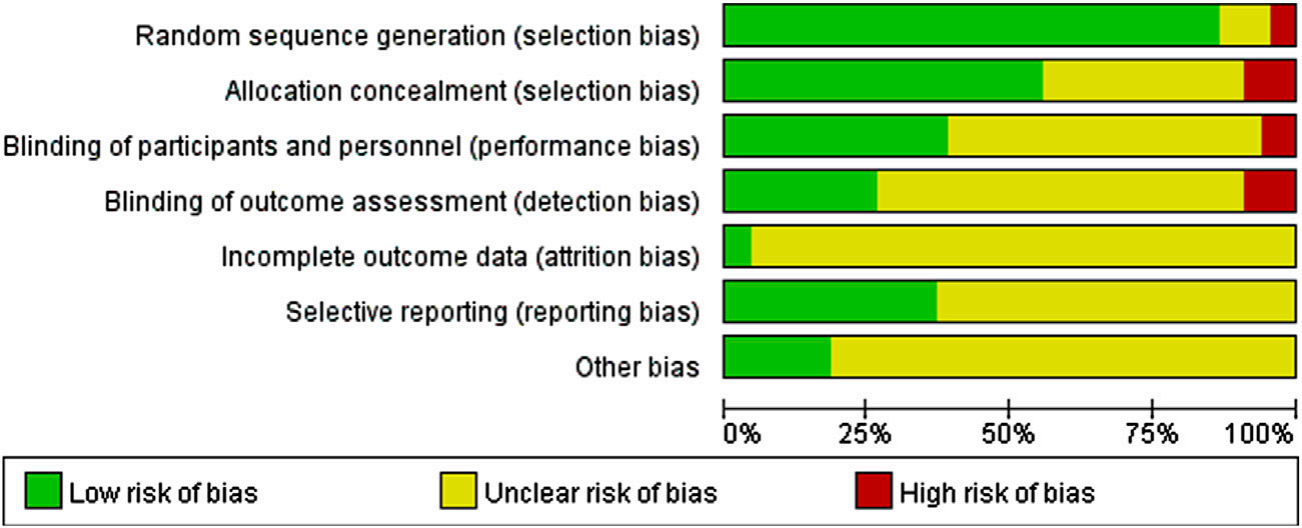
Proportion of items that generated risk of bias in the included literature.

**FIGURE 3:**
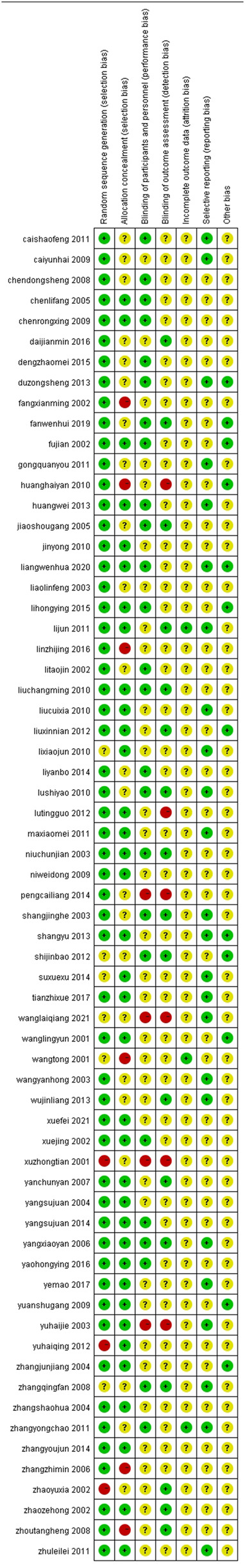
The proportion of items with bias risks in each literature work.

Among the 65 included studies, 56 studies were low-risk, 6 studies only mentioned randomization, but did not specify the random allocation method, rated as unclear risk, and 3 studies did not mention it. In terms of allocation concealment, 38 studies were rated as low risk, 6 studies did not give clear allocation as high risk, and the remaining 23 studies did not mention allocation concealment as unclear risk. In terms of research blinding, 25 studies mentioned it, 36 studies did not mention it, and 4 studies were rated as high risk because they could not be blinded due to different types of interventions included in the studies. In terms of completeness of outcome indicators, 17 items clearly stated that there was no loss of follow-up and were rated as low risk. Six items mentioned loss of follow-up, which was rated as high risk. The remaining 42 did not mention loss to follow-up and were rated as unclear risk. Data were reported selectively; 24 were low-risk and 41 were high-risk. Other risks of bias were only mentioned in 12 articles, and the rest were unclear. The risk of bias assessment of the included studies is shown in Figures 2, 3.

### 3.4 Results

#### 3.4.1 Total clinical effective rate

**FIGURE 4:**
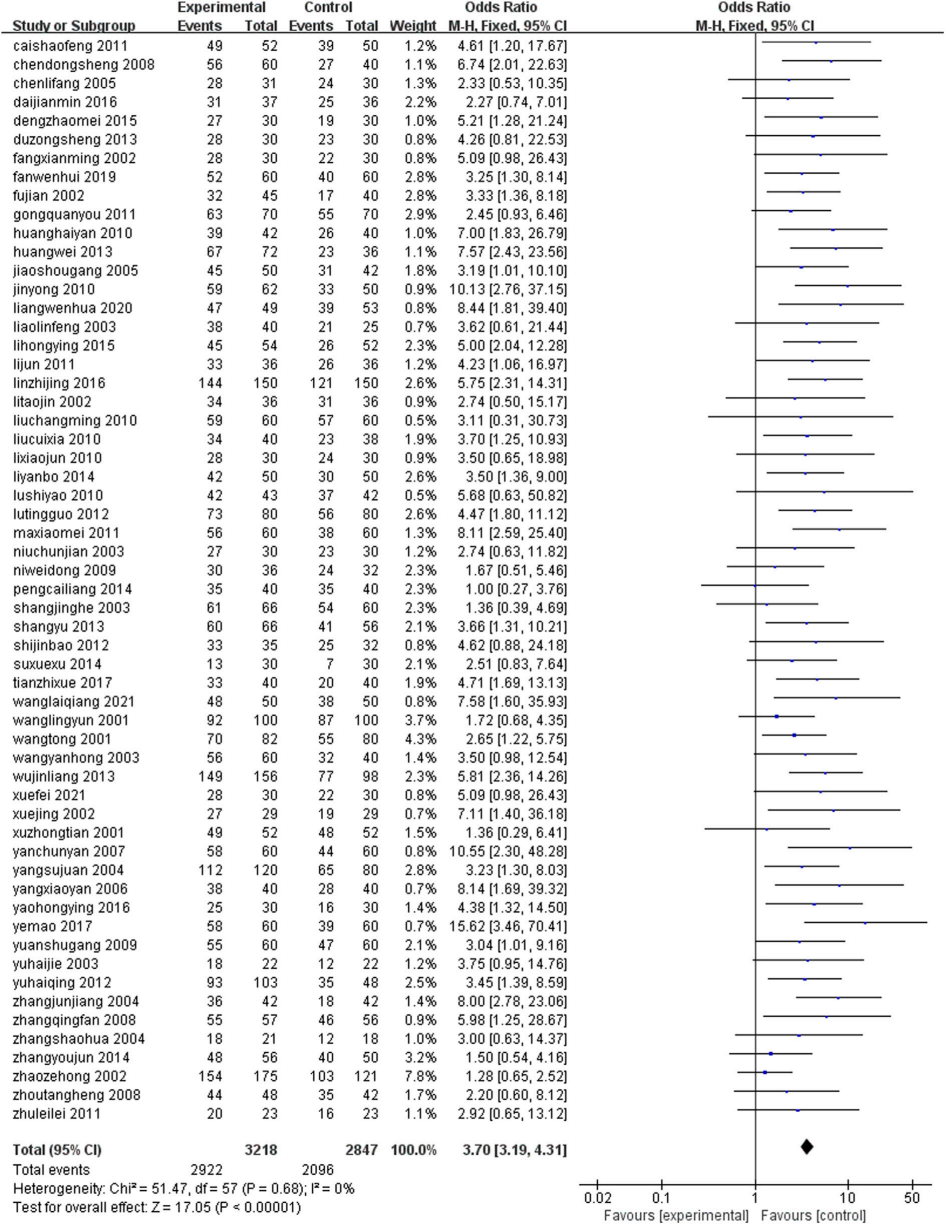
Total effective rate.

A total of 58 studies (Wu et al., 2001; Tong et al., 2001; Zhongtian and Rihui, 2001; Jian and Xudong, 2002; Jing et al., 2002; Taojin, 2002; Xianming et al., 2002; Zehong et al., 2002; Chunjian et al., 2003; Haijie and Changshi, 2003; Jinghe, 2003; Linfeng, 2003; Yanhong et al., 2003; Junjiang et al., 2004; Shaohua, 2004; Sujuan and Haijun, 2004; Lifang and Guofeng, 2005; Shougang, 2005; Xiaoyan and Bainian, 2006; Chunyan, 2007; Dongsheng and Jimin, 2008; Qingfan and Hezhong, 2008; Tangheng, 2008; Shugang et al., 2009; Weidong and Lihua, 2009; Changming, 2010; Cuixia and Cunji, 2010; Haiyan, 2010; Shiyao, 2010; Xiaojun, 2010; Yong, 2010; Jun et al., 2011; Leilei, 2011; Quanyou, 2011; Shaofeng and Xinfeng, 2011; Xiao-Mei, 2011; Haiqing, 2012; Jinbao et al., 2012; Tingguo et al., 2012; Jinliang and Yunyi, 2013; Wei et al., 2013; Yu, 2013; Zongsheng, 2013; Cailiang et al., 2014; Yanbo, 2014; Youjun, 2014; Xuexu et al., 2014; Hongying et al., 2015; Zhaomei and Lian, 2015; Zhijing et al., 2016; Hongying et al., 2016; Jianmin et al., 2016; Mao and Qiang, 2017; Zhixue et al., 2017; Wenhui and Shiliang, 2019; Wenhua et al., 2020; Fei, 2021; Laiqiang, 2021) with total clinical response rate as the outcome index, a total of 6,065 patients were included. The total response rate of 58 studies was homogeneous (P = 0.68, I^2^ = 0%), and a fixed effect model was set. Meta-analysis results showed that: The total effective rate of the treatment group was higher than that of the control group, and the difference was statistically significant [OR = 3.70, 95%CI (3.19, 4.31), Z = 17.05, P < 0.00001], indicating that in terms of improving the total effective rate of coronary heart disease, the combination of conventional western medicine with leech Chinese patent medicine was significantly better than the simple conventional western medicine (Figure 4).

#### 3.4.2 ECG efficacy

**FIGURE 5:**
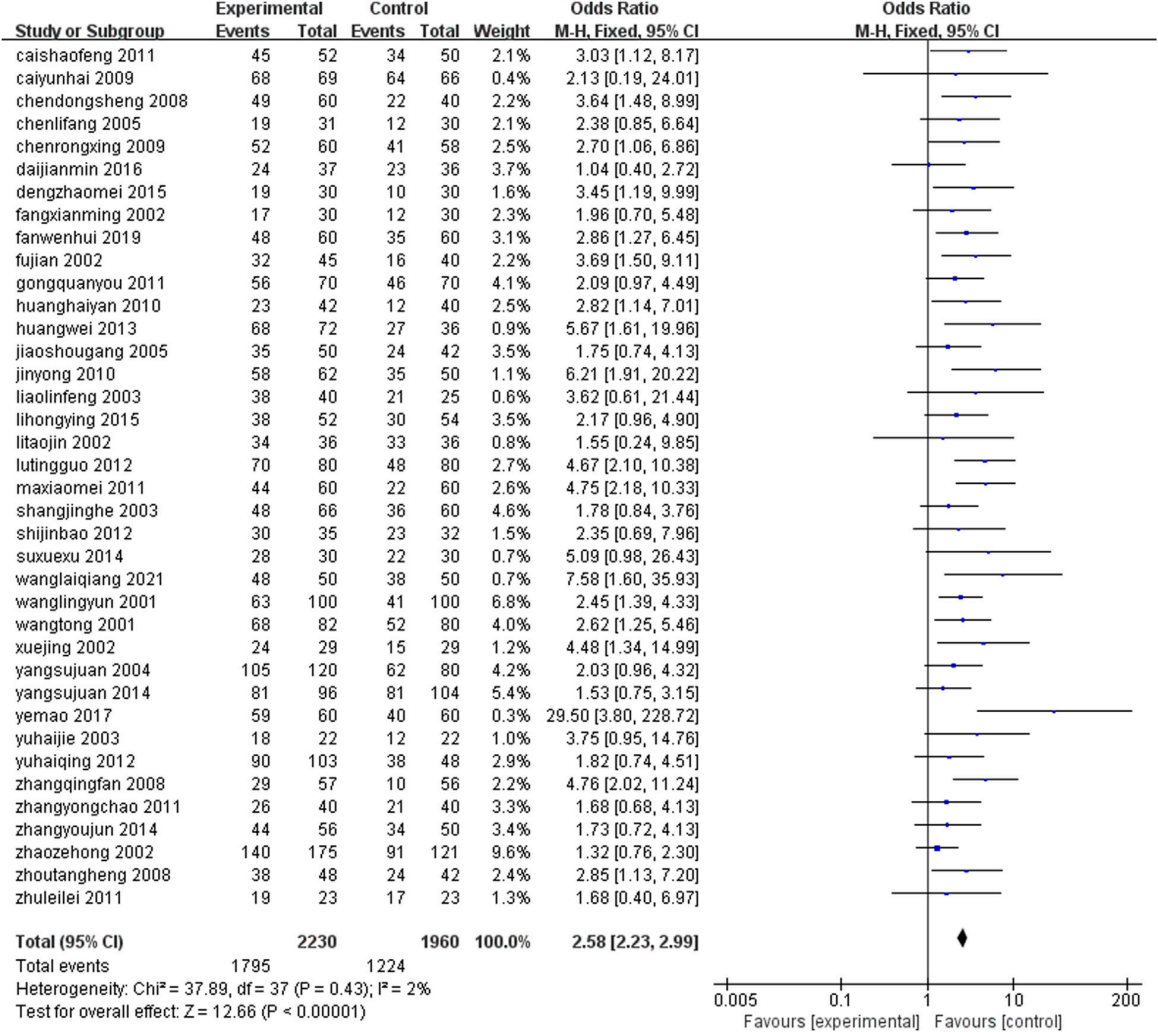
Comparison of ECG efficacy between leech combined with conventional treatment and conventional treatment alone.

Thirty eight studies (Wu et al., 2001; Tong et al., 2001; Jian and Xudong, 2002; Jing et al., 2002; Taojin, 2002; Xianming et al., 2002; Zehong et al., 2002; Haijie and Changshi, 2003; Jinghe, 2003; Linfeng, 2003; Sujuan and Haijun, 2004; Lifang and Guofeng, 2005; Shougang, 2005; Dongsheng and Jimin, 2008; Qingfan and Hezhong, 2008; Tangheng, 2008; Rongxing and Linlin, 2009; Yunhai, 2009; Haiyan, 2010; Yong, 2010; Leilei, 2011; Quanyou, 2011; Shaofeng and Xinfeng, 2011; Xiao-Mei, 2011; Yongchao et al., 2011; Haiqing, 2012; Jinbao et al., 2012; Tingguo et al., 2012; Wei et al., 2013; Sujuan et al., 2014; Youjun, 2014; Xuexu et al., 2014; Hongying et al., 2015; Zhaomei and Lian, 2015; Jianmin et al., 2016; Mao and Qiang, 2017; Wenhui and Shiliang, 2019; Laiqiang, 2021) (n = 4,190) reported the ECG efficacy after treatment. The results of meta-analysis under fixed effects model (I^2^ = 2%, P < 0.0001) showed that the group containing leech Chinese medicine combined with conventional treatment had better ECG efficacy after treatment, and the difference was statistically significant [OR = 2.58, 95%CI (2.23, 2.99), P < 0.0001] (Figure 5).

#### 3.4.3 TCM symptoms and efficacy

**FIGURE 6:**
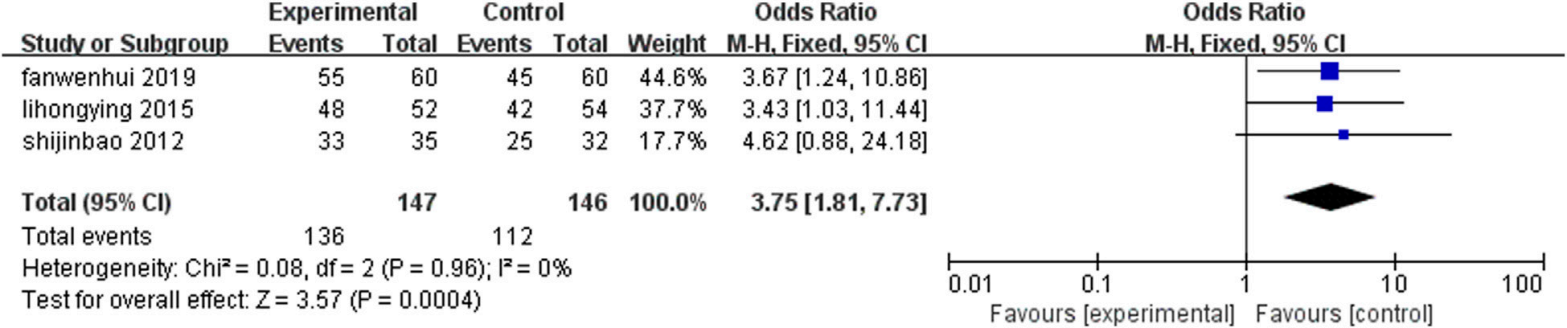
Comparison of the efficacy of leech combined with conventional treatment and conventional treatment alone for TCM symptoms.

Three studies (Jinbao et al., 2012; Hongying et al., 2015; Wenhui and Shiliang, 2019) (n = 293) reported the efficacy of TCM symptoms after treatment, and the difference between the two groups was statistically significant [OR = 3.75, 95%CI (1.81, 7.73), Z = 3.57, P = 0.0004]. The Chinese patent medicine containing leech combined with conventional medicine in the treatment of CHD could improve the efficacy of TCM symptoms than conventional western medicine alone (Figure 6).

#### 3.4.4 Frequency of angina pectoris

**FIGURE 7:**
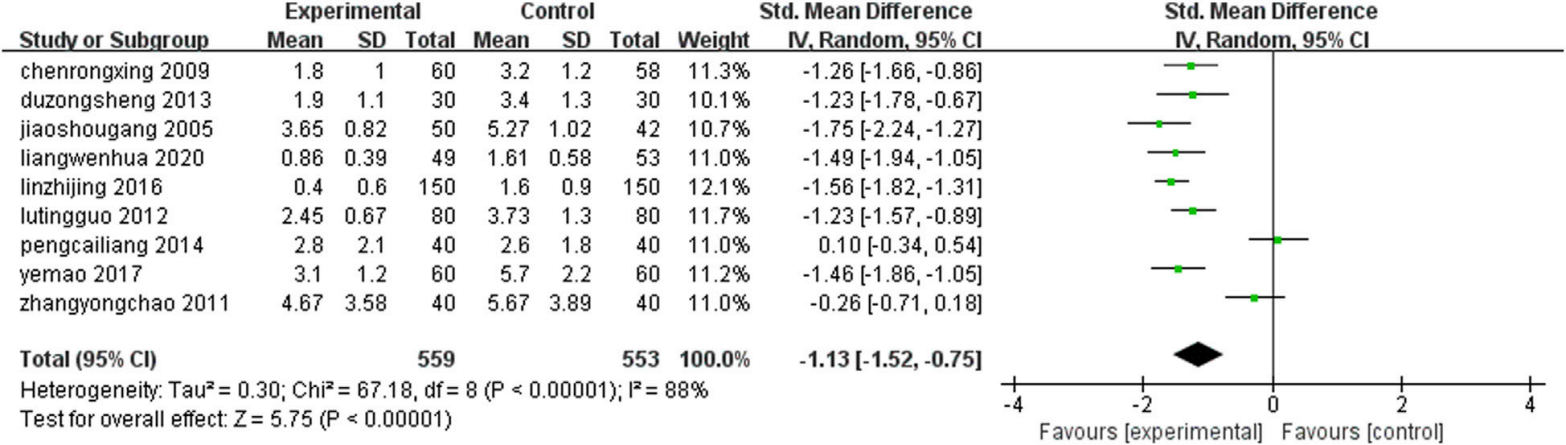
Comparison of frequency of angina episodes between leech combined with conventional treatment and conventional treatment alone.

Nine studies (Shougang, 2005; Rongxing and Linlin, 2009; Yongchao et al., 2011; Tingguo et al., 2012; Zongsheng, 2013; Cailiang et al., 2014; Zhijing et al., 2016; Mao and Qiang, 2017; Wenhua et al., 2020) (n = 1,112) reported the frequency of angina pectoris in each group after treatment. The results of Meta-analysis showed that the frequency of angina pectoris attack after treatment with leech Chinese medicine combined with conventional medication was lower than that of conventional treatment alone, and the incidence of angina pectoris was significantly reduced. The difference between the two groups was statistically significant [SMD = −1.13, 95%CI (-1.52, −0.75), Z = 5.75, P ≤ 0.00001] (Figure 7).

#### 3.4.5 Whole blood viscosity

**FIGURE 8:**
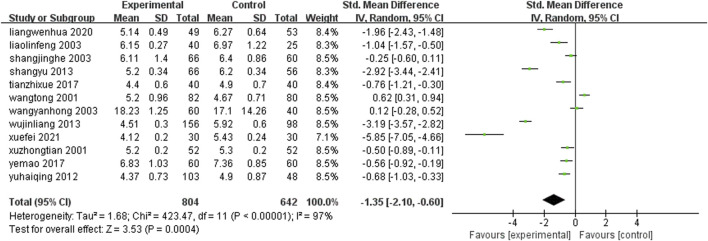
Comparison of whole blood viscosity between leech combined with conventional treatment and conventional treatment alone.

Twelve studies (Tong et al., 2001; Zhongtian and Rihui, 2001; Jinghe, 2003; Linfeng, 2003; Yanhong et al., 2003; Haiqing, 2012; Jinliang and Yunyi, 2013; Yu, 2013; Mao and Qiang, 2017; Zhixue et al., 2017; Wenhua et al., 2020; Fei, 2021) (n = 1,446) reported on post-treatment whole blood viscosity levels in each group. Meta-analysis results showed that: The whole blood viscosity level was lower after the treatment of Chinese patent medicine containing leech ingredients combined with conventional medication than that of conventional treatment alone, and the difference between the two groups was statistically significant [SMD = −1.35, 95%CI (−2.10, −0.60), Z = 3.53, P = 0.0004] (Figure 8).

#### 3.4.6 Plasma viscosity

**FIGURE 9:**
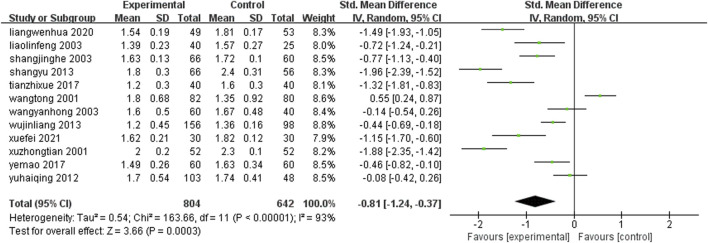
Comparison of plasma viscosity between leech combined with conventional treatment and conventional treatment alone.

Blood urea nitrogen levels 12 studies (Tong et al., 2001; Zhongtian and Rihui, 2001; Jinghe, 2003; Linfeng, 2003; Yanhong et al., 2003; Haiqing, 2012; Jinliang and Yunyi, 2013; Yu, 2013; Mao and Qiang, 2017; Zhixue et al., 2017; Wenhua et al., 2020; Fei, 2021) (n = 1,446) reported changes in plasma viscosity after treatment in each group. Meta-analysis results showed that: The plasma viscosity level after treatment with leech Chinese proprietary medicine combined with conventional medication was lower than that of conventional treatment alone, and the difference was statistically significant [SMD = −0.81, 95%CI(-1.24, −0.37), Z = 3.66, P = 0.0003] (Figure 9).

#### 3.4.7 Fibrinogen

**FIGURE 10:**
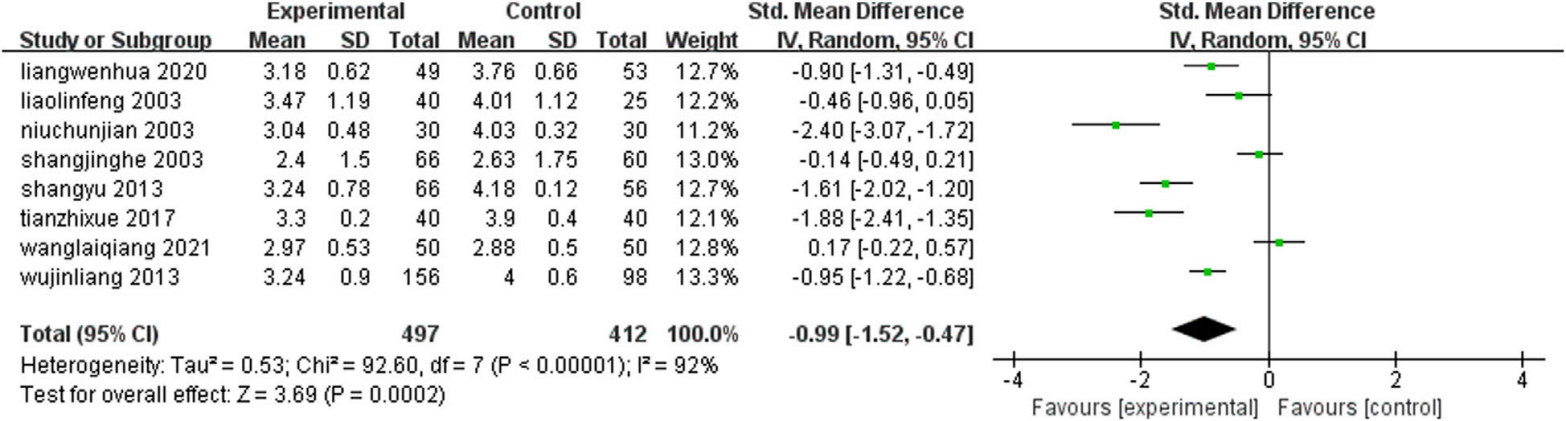
Comparison of leech combined with conventional treatment and conventional treatment alone for fibrinogen.

Eight studies (Chunjian et al., 2003; Jinghe, 2003; Linfeng, 2003; Jinliang and Yunyi, 2013; Yu, 2013; Zhixue et al., 2017; Wenhua et al., 2020; Laiqiang, 2021) (n = 909) reported fibrinogen levels in each group after treatment. Meta-analysis results showed that: The level of fibrinogen in the treatment group was lower than that in the control group [SMD = −0.99, 95%CI (−1.52, −0.47), Z = 3.69, P = 0.0002] (Figure 10).

#### 3.4.8 Total cholesterol

**FIGURE 11:**
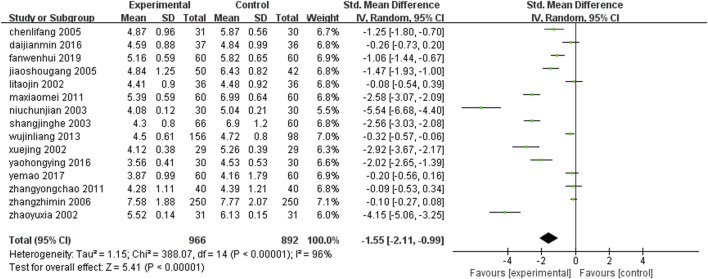
Comparison of total cholesterol between leech combined with conventional treatment and conventional treatment alone.

Fifteen studies (Jing et al., 2002; Taojin, 2002; Yuxia et al., 2002; Chunjian et al., 2003; Jinghe, 2003; Lifang and Guofeng, 2005; Shougang, 2005; Zhi-Min et al., 2006; Xiao-Mei, 2011; Yongchao et al., 2011; Jinliang and Yunyi, 2013; Hongying et al., 2016; Jianmin et al., 2016; Mao and Qiang, 2017; Wenhui and Shiliang, 2019) (n = 1858) reported the serum total cholesterol levels of each group after treatment. The results of Meta-analysis showed that the serum total cholesterol level after treatment with leech Chinese medicine combined with conventional treatment was lower than that of conventional treatment alone. The difference between the two groups was statistically significant [SMD = −1.55, 95%CI (−2.11, −0.99), Z = 5.41, P ≤ 0.00001] (Figure 11).

#### 3.4.9 Triglycerides

**FIGURE 12:**
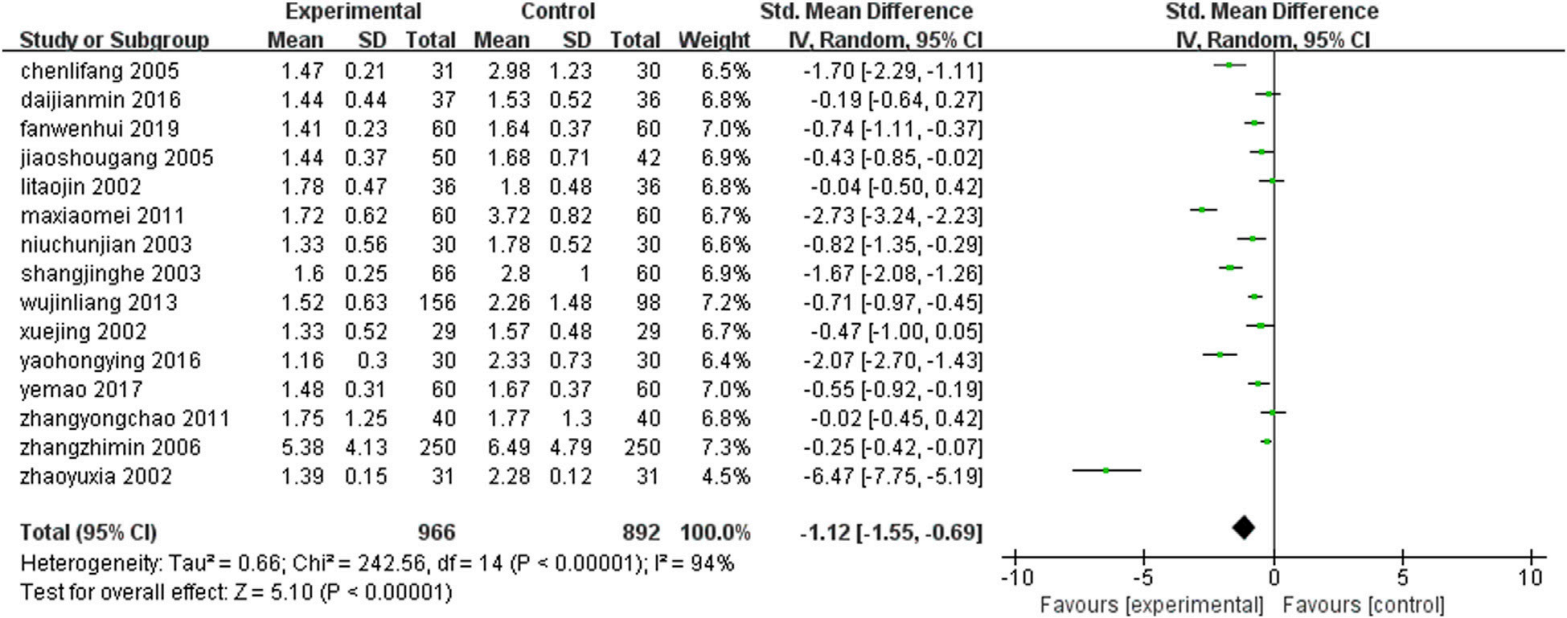
Comparison of leeches combined with conventional treatment and conventional treatment alone triglyceride.

Fifteen studies (Jing et al., 2002; Taojin, 2002; Yuxia et al., 2002; Chunjian et al., 2003; Jinghe, 2003; Lifang and Guofeng, 2005; Shougang, 2005; Zhi-Min et al., 2006; Xiao-Mei, 2011; Yongchao et al., 2011; Jinliang and Yunyi, 2013; Hongying et al., 2016; Jianmin et al., 2016; Mao and Qiang, 2017; Wenhui and Shiliang, 2019) (n = 1858) reported the serum triglyceride levels of each group after treatment. The results of Meta-analysis showed that the serum triglyceride level of the treatment with leech Chinese medicine combined with conventional treatment was lower than that of conventional treatment alone. The difference between the two groups was statistically significant [SMD = −1.12, 95%CI (−1.55, −0.69), Z = 5.10, P ≤ 0.00001] (Figure 12).

#### 3.4.10 High density lipoprotein

**FIGURE 13:**
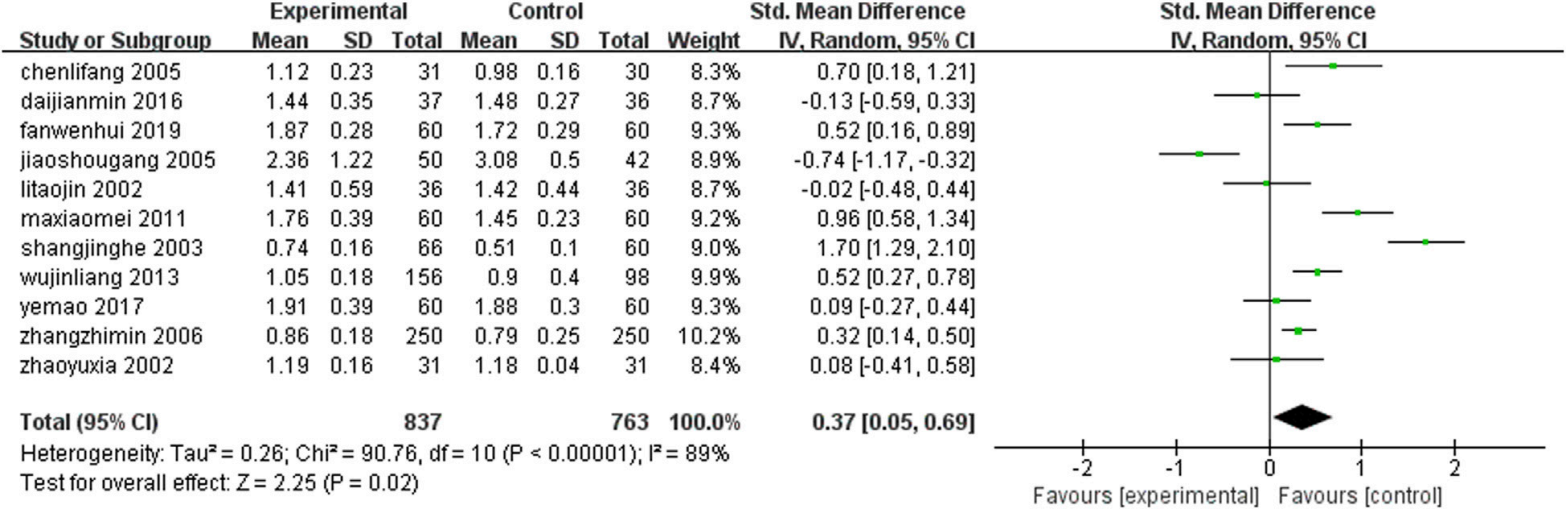
Comparison of leech combined with conventional treatment and conventional treatment alone for HDL.

Eleven studies (Taojin, 2002; Yuxia et al., 2002; Jinghe, 2003; Lifang and Guofeng, 2005; Shougang, 2005; Zhi-Min et al., 2006; Xiao-Mei, 2011; Jinliang and Yunyi, 2013; Jianmin et al., 2016; Mao and Qiang, 2017; Wenhui and Shiliang, 2019) (n = 1,600) reported changes in HDL after treatment in each group. Meta-analysis results showed that: The level of high-density lipoprotein after leech combined with conventional medication was higher than that of conventional treatment alone, but there was no statistical significance [SMD = 0.37, 95%CI (0.05, 0.69), Z = 2.25, P = 0.02] (Figure 13).

#### 3.4.11 Low density lipoprotein

**FIGURE 14:**
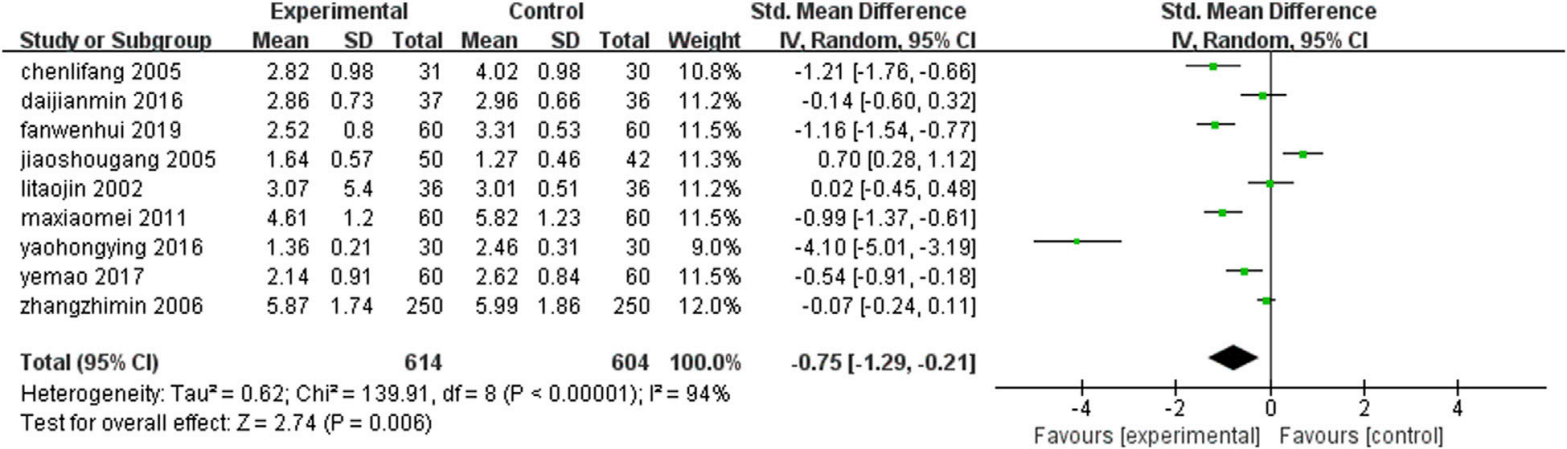
Leech combined with conventional treatment compared with conventional treatment alone for LDL.

Nine studies (Taojin, 2002; Lifang and Guofeng, 2005; Shougang, 2005; Zhi-Min et al., 2006; Xiao-Mei, 2011; Hongying et al., 2016; Jianmin et al., 2016; Mao and Qiang, 2017; Wenhui and Shiliang, 2019) (n = 1,218) reported the levels of low-density lipoprotein in each group after treatment. The results of Meta-analysis showed that the level of low-density lipoprotein after treatment with leech Chinese medicine combined with conventional treatment was lower than that of conventional treatment alone. The difference between the two groups was statistically significant [SMD = −0.75, 95%CI (−1.29, −0.21), Z = 2.74, P < 0.006] (Figure 14).

#### 3.4.12 Adverse reactions

**FIGURE 15:**
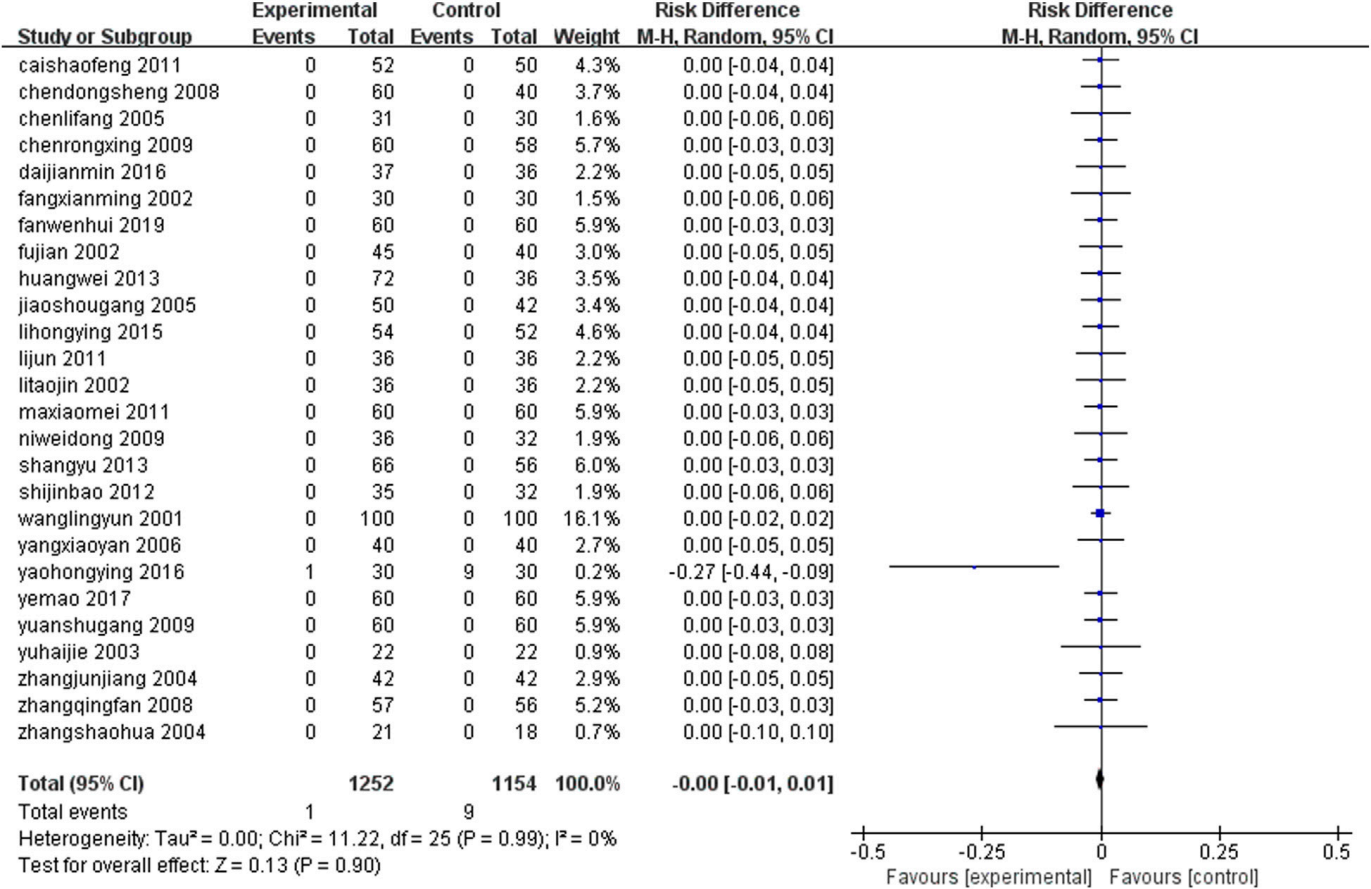
Adverse reactions.

Twenty six studies (Wu et al., 2001; Jian and Xudong, 2002; Taojin, 2002; Xianming et al., 2002; Haijie and Changshi, 2003; Junjiang et al., 2004; Shaohua, 2004; Lifang and Guofeng, 2005; Shougang, 2005; Xiaoyan and Bainian, 2006; Dongsheng and Jimin, 2008; Qingfan and Hezhong, 2008; Rongxing and Linlin, 2009; Shugang et al., 2009; Weidong and Lihua, 2009; Jun et al., 2011; Shaofeng and Xinfeng, 2011; Xiao-Mei, 2011; Jinbao et al., 2012; Wei et al., 2013; Yu, 2013; Hongying et al., 2015; Hongying et al., 2016; Jianmin et al., 2016; Mao and Qiang, 2017; Wenhui and Shiliang, 2019) (n = 2,406) reported the adverse reactions that occurred in the patients of each group after treatment. The number of adverse reactions and their symptoms are presented in Table 3. All adverse reactions were mild and resolved spontaneously after treatment. All adverse reactions were effectively managed through regular monitoring and appropriate intervention. The study found that compared with the group that only received conventional treatment, the incidence of adverse events in the group that received combined treatment with herbal medicine from earthworms did not significantly increase. The results of the meta-analysis showed that there was no statistically significant difference in the incidence of adverse reactions between the two groups [RD = 0.00, 95%CI (−0.01, 0.01), P = 0.90] (Figure 15).

**TABLE 3:** Adverse reaction symptom.

Adverse reaction			
Author	Year	Number	Symptoms
Yao hong ying	2016	1	Mild vomiting
Li hong ying	2015	1	Mild thirst
Ma xiao mei	2011	3	Slight headache
Liu cui xia	2010	2	Minor bleeding on the skin
Yuan shu gang	2009	1	Slight headache
Chen rong xing	2009	1	Gastric discomfort, relieved by eating
Yang xiao yan	2006	1	Gastric discomfort, relieved by eating
Fu jian	2002	3	Gastric discomfort, relieved by eating
Wang ling yun	2001	1	Gastrointestinal discomfort

### 3.5 Subgroup analysis

**FIGURE 16:**
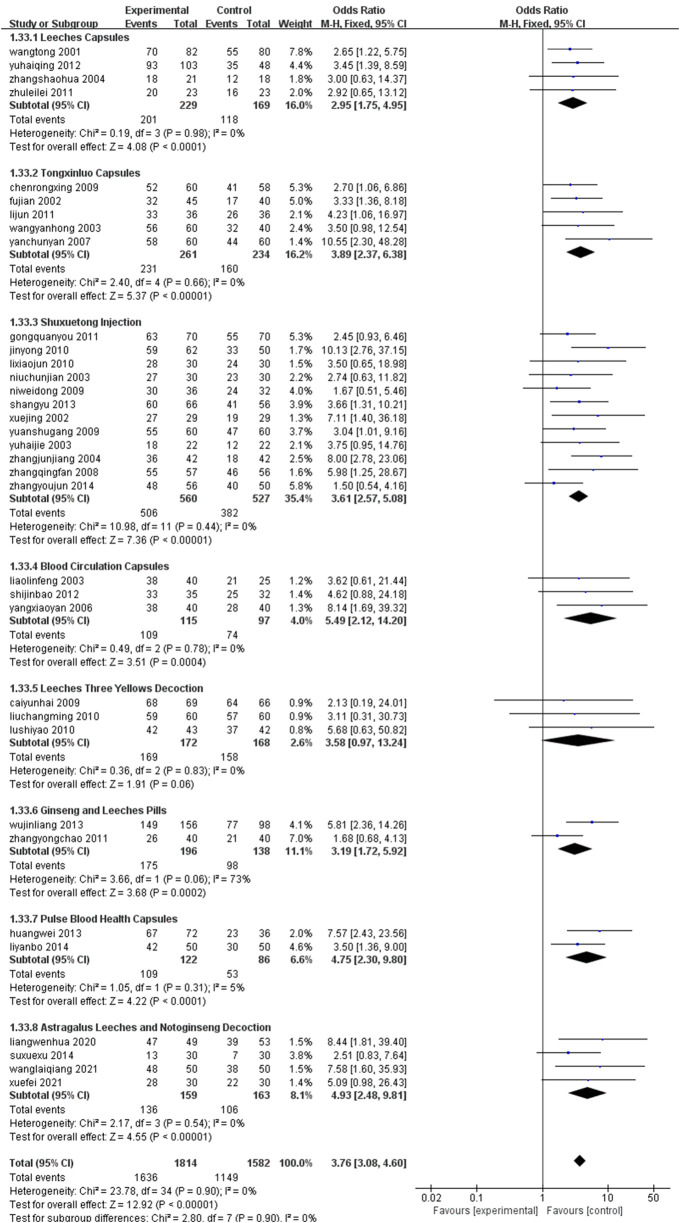
Drug classification grouping.

**FIGURE 17:**
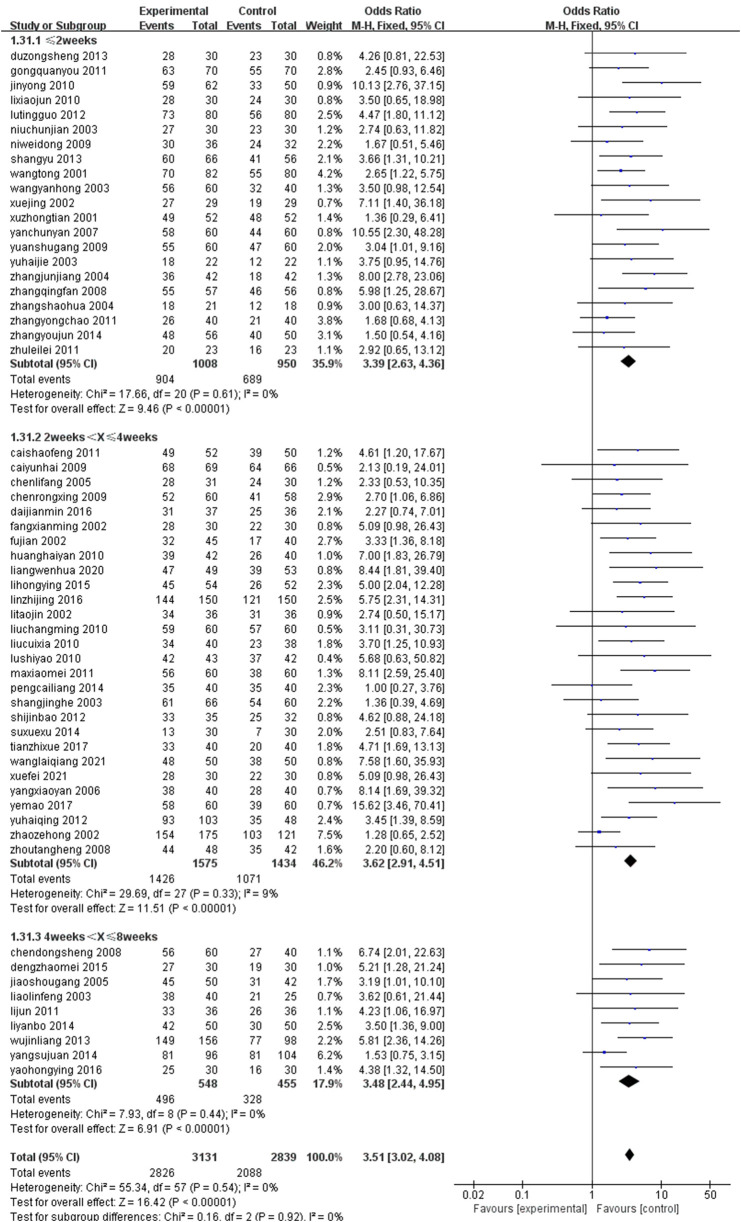
Treatment time grouping.

Subgroup analyses were performed on specific outcomes based on two primary intervention factors: drug type and treatment duration. The results consistently demonstrated that the experimental treatment outperformed the control group. The overall pooled odds ratio for all treatment combinations across subgroups classified by drug type was 3.76 (95% CI: 3.08–4.60), indicating a robust and consistent effect of the experimental treatment across all subgroups. Similarly, the overall effect across all treatment durations in the subgroups stratified by treatment duration was 3.51 (95% CI: 3.02–4.08), suggesting that treatment duration did not significantly impact the results, with the experimental treatment remaining effective across various timeframes. Statistical significance was observed in all subgroups. The forest plots of the results are presented in Figures 16, 17.

### 3.6 Publication bias analysis

**FIGURE 18:**
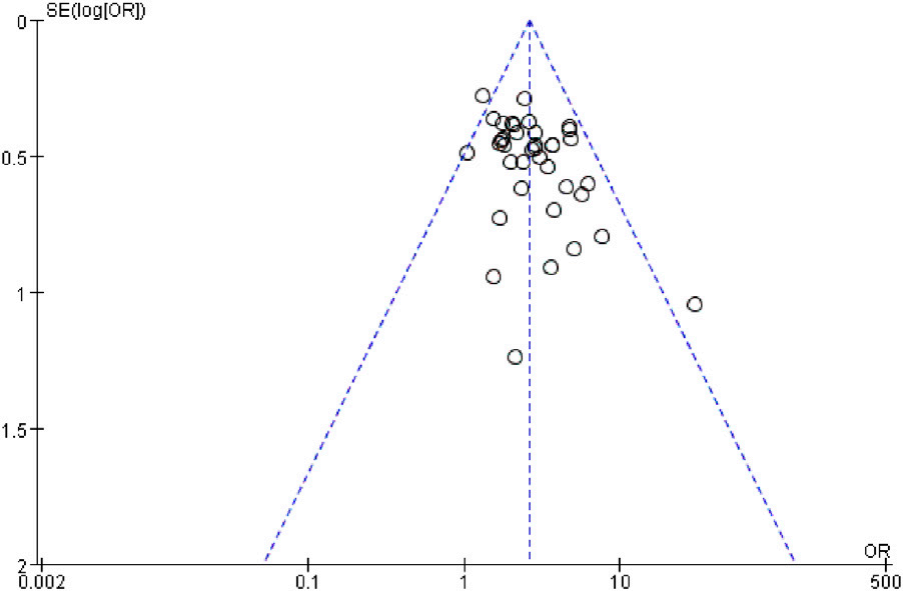
Funnel plot of total clinical response rate.

**FIGURE 19:**
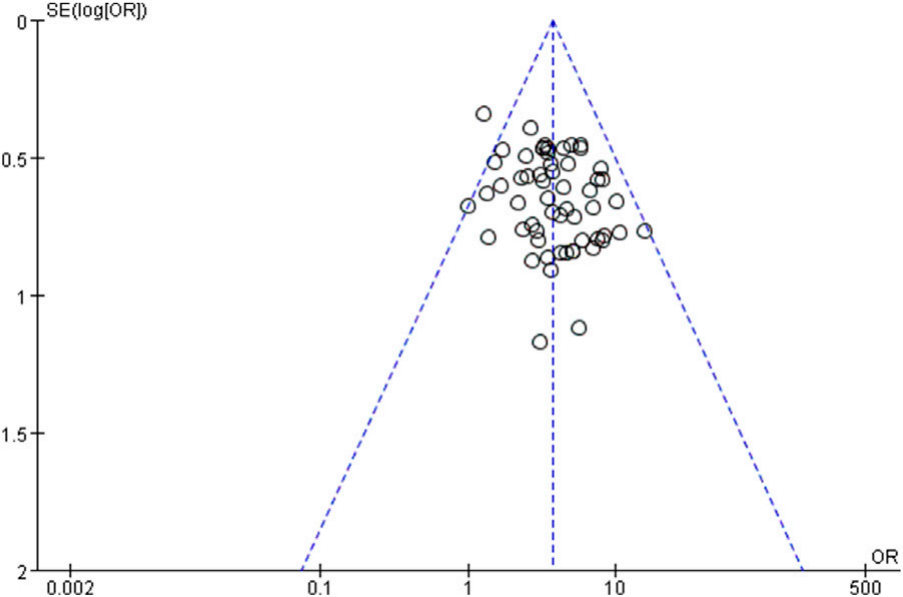
Funnel plot of ECG efficacy.

The risk of publication bias was evaluated using funnel plots for the total effective rate and ECG efficacy of the primary outcome indicators, as shown in Figures 18, 19, respectively. It was observed that the scatter points of the funnel plot of the two outcome indicators were distributed above the horizontal axis, but the distribution was basically symmetrical, suggesting that there was no risk of publication bias.

### 3.7 GRADE assessment

Using the GRADE approach, we conducted a certainty assessment for the primary outcome measures. For effective and ECG efficacy, the evidence was downgraded by one level due to serious concerns about risk of bias, resulting in moderate certainty of evidence (Table 4). For traditional Chinese medicine symptom efficacy, the evidence was rated as moderate certainty due to a high risk of bias. For the frequency of angina pectoris, the evidence was downgraded by two levels due to both a high risk of bias and publication bias, leading to low-certainty evidence (Table 5). For whole blood viscosity, plasma viscosity, and fibrinogen, some studies had limitations in clinical applicability, high uncertainty in results, and uncontrolled confounding factors, resulting in low-certainty evidence (Table 6). Regarding the biochemical indicator total cholesterol, although there were significant inconsistencies and precision issues, the risk of bias was low, and the evidence was rated as moderate certainty. For triglycerides, considering the substantial inconsistencies, precision issues, and the potential impact of residual confounding factors, the evidence was downgraded by two levels, ultimately rated as low certainty. For high-density lipoprotein (HDL), despite some inconsistencies and precision issues, the risk of bias was low, and the evidence was rated as moderate certainty. For low-density lipoprotein (LDL), due to severe inconsistencies, precision issues, and substantial publication bias, the evidence was downgraded by two levels, resulting in very low-certainty evidence (Table 7).

**TABLE 4:** Effective and ECG efficacy assessment.

Certainty assessment							No. of patients		Effect		Certainty	Importance
No. of studies	Study design	Risk of bias	Inconsistency	Indirectness	Imprecision	Other considerations	New comparison	Placebo	Relative (95% CI)	Absolute (95% CI)		
Effective												
58	Randomised trials	Serious	Not serious	Not serious	Not serious	None	2922/3218 (90.8%)	2096/2847 (73.6%)	**OR 3.70** (3.19 to 4.31)	**175 more per 1,000** (from 163 more to 187 more)	⨁⨁⨁○ Moderate	CRITICAL
Electrocardiogram efficacy												
38	Randomised trials	Serious	Not serious	Not serious	Not serious	None	1795/2230 (80.5%)	1224/1960 (62.4%)	**OR 2.58** (2.23 to 2.99)	**186 more per 1,000** (from 163 more to 208 more)	⨁⨁⨁○ Moderate	CRITICAL

CI, confidence interval; MD, mean difference; OR, odds ratio.

**TABLE 5:** TCM symptoms and frequency of angina pectoris.

Certainty assessment							No. of patients		Effect		Certainty	Importance
No. of studies	Study design	Risk of bias	Inconsistency	Indirectness	Imprecision	Other considerations	New comparison	Placebo	Relative (95% CI)	Absolute (95% CI)		
Traditional Chinese medicine, Symptom, Efficacy (二)												
3	Randomised trials	Serious	Not serious	Not serious	Not serious	None	136/147 (92.5%)	112/146 (76.7%)	**OR 3.75** (1.81 to 7.73)	**158 more per 1,000** (from 89 more to 195 more)	⨁⨁⨁○ Moderate	IMPORTANT
Frequency of angina pectoris												
9	Randomised trials	Serious	Very serious	Not serious	Not serious	All plausible residual confounding would reduce the demonstrated effect	559	553	-	**MD 1.14 lower** (1.25 lower to 1.04 lower)	⨁⨁○○ Low	IMPORTANT

CI, confidence interval; MD, mean difference; OR, odds ratio

**TABLE 6:** Whole blood viscosity, plasma viscosity, and fibrinogen.

Certainty assessment							No. of patients		Effect		Certainty	Importance
No. of studies	Study design	Risk of bias	Inconsistency	Indirectness	Imprecision	Other considerations	New comparison	Placebo	Relative (95% CI)	Absolute (95% CI)		
Whole blood viscosity												
12	Randomised trials	Not serious	Serious	Serious	Serious	All plausible residual confounding would reduce the demonstrated effect	804	642	-	**MD 0.69 lower** (0.73 lower to 0.64 lower)	⨁⨁○○ Low	IMPORTANT
Plasma viscosity												
12	Randomised trials	Not serious	Serious	Not serious	Serious	None	804	642	-	**MD 0.19 lower** (0.22 lower to 0.17 lower)	⨁⨁○○ Low	IMPORTANT
Fibrinogen												
8	Randomised trials	Not serious	Serious	Not serious	Serious	None	497	412	-	**MD 0.63 lower** (0.7 lower to 0.55 lower)	⨁⨁○○ Low	IMPORTANT

CI, confidence interval; MD, mean difference; OR, odds ratio.

**TABLE 7:** Total cholesterol triglycerides HDL and LDL.

Certainty assessment							No. of patients		Effect		Certainty	Importance
No. of studies	Study design	Risk of bias	Inconsistency	Indirectness	Imprecision	Other considerations	New comparison	Placebo	Relative (95% CI)	Absolute (95% CI)		
Total cholesterol												
15	Randomised trials	Not serious	Serious	Not serious	Serious	All plausible residual confounding would reduce the demonstrated effect	966	892	-	**MD 0.79 lower** (0.84 lower to 0.75 lower)	⨁⨁⨁○ Moderate	IMPORTANT
Triglycerides												
15	randomised trials	not serious	very serious	not serious	serious	all plausible residual confounding would reduce the demonstrated effect	966	892	-	**MD 0.62 lower** (0.67 lower to 0.58 lower)	⨁⨁○○ Low	IMPORTANT
High density lipoprotein												
11	Randomised trials	Not serious	Serious	Serious	Not serious	All plausible residual confounding would reduce the demonstrated effect	837	763	-	**MD 0.11 higher** (0.09 higher to 0.14 higher)	⨁⨁⨁○ Moderate	IMPORTANT
Low density lipoprotein												
9	Randomised trials	Not serious	Very serious	Not serious	Serious	Publication bias strongly suspected all plausible residual confounding would reduce the demonstrated effect	614	604	-	**MD 0.63 lower** (0.71 lower to 0.54 lower)	⨁○○○ Very low	IMPORTANT

## 4 Discussion

According to the results of this meta-analysis, Chinese patent medicine containing leech has shown clinical efficacy in the treatment of coronary heart disease, especially in improving the overall cure rate, abnormal electrocardiogram, hemorheology indexes and TCM symptom scores. The treatment group was superior to conventional western medicine in reducing the frequency of angina pectoris, improving the ischemic changes of electrocardiogram and regulating blood lipid levels. This may elucidate the effective effects of leech components on relieving angina pectoris and improving myocardial ischemia can be clarified.

The results of this meta-analysis show that leech-based treatments significantly improved the total effective rate and ECG outcomes in CHD patients. Specifically, the treatment group exhibited better clinical responses compared to those receiving only conventional treatments. This is crucial, as CHD patients typically suffer from ischemic changes reflected in abnormal ECG results (Albus et al., 2017; Su et al., 2023). The improvement in ECG outcomes suggests that hirudin play a role in mitigating myocardial ischemia. Hirudin, by inhibiting thrombin, directly reduces thrombosis and platelet aggregation, thereby enhancing coronary blood flow (Junren et al., 2021). This improvement likely contributes to the observed reduction in ischemic changes on the ECG.

Another critical finding is the significant reduction in the frequency of angina pectoris attacks in the treatment group, alongside improvements in hemorheological parameters whole blood and plasma viscosity. These results suggest that leech-based treatments contribute to better blood flow, particularly in coronary microcirculation. The reduction in blood viscosity, facilitated by hirudin’s anticoagulant properties, enhances tissue perfusion, ensuring that oxygen and nutrients reach ischemic myocardial tissue more efficiently. This is consistent with the known mechanisms of hirudin, which improves blood fluidity and prevents clot formation (Junren et al., 2021; Qiu et al., 2022), thus reducing the incidence of angina attacks and alleviating myocardial ischemia.

The meta-analysis also demonstrated significant improvements in lipid profiles, including reductions in total cholesterol, LDL, and triglycerides, along with a modest increase in HDL. These lipid changes are important for the long-term management of CHD, as they help slow the progression of atherosclerosis (Kamstrup, 2021; Shaya et al., 2022; Alloubani et al., 2021; Humm et al., 2023; Ueki et al., 2024) By improving lipid metabolism, substances in leeches may help stabilize atherosclerotic plaques, reducing the risk of plaque rupture and subsequent cardiovascular events. The observed lipid improvements may be attributed to the anti-inflammatory effects of leech components, which can reduce lipid oxidation and promote healthier vascular function.

The clinical benefits observed in this study are closely tied to the pharmacological properties ofsubstances in leeches. Hirudin, as a potent anticoagulant, plays a central role in preventing thrombus formation and improving blood flow (Junren et al., 2021). Its ability to reduce fibrinogen levels and enhance blood fluidity is crucial in alleviating myocardial ischemia. Moreover, the reduction in blood viscosity enhances coronary microcirculation, leading to better tissue perfusion and a reduction in ischemic damage to the heart (Del Buono et al., 2021).

Additionally, the improvement in lipid metabolism, evidenced by the decrease in LDL and increase in HDL, is likely due to the anti-inflammatory and antioxidant properties of leech components (Junren et al., 2021). These effects help reduce oxidative stress and inflammation, which are key factors in the progression of atherosclerosis (Kong et al., 2022; Attiq et al., 2024; Violi et al., 2024; Guo et al., 2024). Moreover, leech components’ ability to promote nitric oxide (NO) release by up-regulating endothelial nitric oxide synthase (eNOS) enhances vascular relaxation, improves coronary perfusion, and contributes to better endothelial function, all of which are essential for managing CHD (Junren et al., 2021; Jaishankar et al., 2023; Sun et al., 2025).

## 5 Limitation

This study has several limitations. Firstly, the quality of some of the included studies was relatively low, particularly regarding randomization, allocation concealment, and blinding, where descriptions were unclear, potentially compromising the reliability of the results. Secondly, some studies had small sample sizes and short follow-up durations, making it difficult to assess the long-term efficacy and safety of the treatment comprehensively. Additionally, there was heterogeneity in the treatment protocols and traditional Chinese medicine formulas used, which could contribute to variations in the results and limit the ability to assess the efficacy of individual treatment plans clearly. Moreover, most of the studies included in this analysis were conducted in China, with the majority of the literature being in Chinese, which may introduce regional and cultural biases, thereby limiting the generalizability of the findings. Despite conducting a thorough literature search, there is still the possibility of publication bias, particularly with respect to the underreporting of negative results. Finally, the existing studies do not provide an in-depth exploration of the specific mechanisms of action of the leech-derived components, the mechanism by which hirudin exerts its effects through improving endothelial function, nitric oxide production, and regulating inflammatory responses is primarily based on existing pharmacological research and theoretical speculation, rather than being directly supported by the clinical data from this study, preventing a full understanding of their precise role in treating coronary heart disease. Special attention must be given to the potential interaction between bioactive components extracted from leeches and conventional anticoagulant or cardiovascular medications. While the therapeutic benefits of leech extracts are evident, combining these extracts with other medications requires careful monitoring of bleeding risks in patients. Some patients may have a history of prior medications or multiple comorbidities, which could influence treatment outcomes. Due to the design and scope of the current study, these factors were not fully accounted for. Although we evaluated several alternative biomarkers in this study, which provide valuable insights into biological effects, it is important to emphasize that these biomarkers cannot directly demonstrate clinical benefit. While they may reflect underlying biological mechanisms or the impact of therapeutic interventions, they are not equivalent to clinical endpoints, such as myocardial infarction, mortality, or quality of life, which are the critical measures for determining the clinical significance of a treatment. Therefore, although alternative biomarkers can serve as useful early predictors, they cannot fully substitute for definitive clinical outcomes. Future research should focus on further exploring these mechanisms, prioritizing clinical endpoints, validating the relationship between alternative biomarkers and actual clinical benefits, and assessing treatment efficacy across diverse patient populations and coronary artery disease subtypes to provide more precise clinical guidance.

## 6 Conclusion

The results of this meta-analysis show that the Chinese patent medicine containing leech has good clinical efficacy in the treatment of coronary heart disease, which can significantly improve the symptoms, electrocardiogram performance and hemorheological indicators of patients, and has high safety. Its multi-target and synergistic mechanism provide a potentially effective TCM treatment for CHD.

Although this study has a certain clinical guiding significance, due to the low quality of some included studies, more high-quality, large sample and long-term follow-up randomized controlled trials are still needed to further verify its efficacy and safety, and clarify its mechanism of action, optimal treatment regimen and applicable population. In general, the potential of Chinese patent medicine containing leech in the comprehensive treatment of coronary heart disease is worthy of further research and application.

## Data Availability

The original contributions presented in the study are included in the article/Supplementary Material, further inquiries can be directed to the corresponding author.

## Abbreviations

CHD, Coronary heart disease; CNKI, China National Knowledge Infrastructure; VIP, VIP China Science and Technology Journal Database; RCTs, Randomized controlled trials; SMD, Standardized Mean Difference; CI, Confidence intervals; OR, Odds Ratio; TCM, Traditional Chinese medicine; RD, Relative risk difference; MD, Mean difference.
