# Supplementary material for: The clinical efficacy of herbal medicines containing leeches in the treatment of coronary heart disease: a systematic review and meta-analysis
Source: Front Pharmacol. 2025 Oct 17;16:1643611. doi: 10.3389/fphar.2025.1643611 (PMC12575325; doi:10.3389/fphar.2025.1643611)
Supplement: Supplementary file 1 [file Table1.docx]

**Author(s):**

**Question:** New Comparison compared to placebo for [health problem]

**Setting:**

**Bibliography:** . [Intervention] for [health problem].

| **Certainty assessment** | | | | | | | **№ of patients** | | **Effect** | | **Certainty** | **Importance** |
| --- | --- | --- | --- | --- | --- | --- | --- | --- | --- | --- | --- | --- |
| **№ of studies** | **Study design** | **Risk of bias** | **Inconsistency** | **Indirectness** | **Imprecision** | **Other considerations** | **New Comparison** | **placebo** | **Relative (95% CI)** | **Absolute (95% CI)** |  |  |
| **effective** | | | | | | | | | | | | |
| 58 | randomised trials | serious | not serious | not serious | not serious | none | 2922/3218 (90.8%) | 2096/2847 (73.6%) | **OR 3.70** (3.19 to 4.31) | **175 more per 1,000** (from 163 more to 187 more) | ⨁⨁⨁◯ Moderate | CRITICAL |
| **Electrocardiogram efficacy** | | | | | | | | | | | | |
| 38 | randomised trials | serious | not serious | not serious | not serious | none | 1795/2230 (80.5%) | 1224/1960 (62.4%) | **OR 2.58** (2.23 to 2.99) | **186 more per 1,000** (from 163 more to 208 more) | ⨁⨁⨁◯ Moderate | CRITICAL |

**CI:** confidence interval; **MD:** mean difference; **OR:** odds ratio
