# Supplementary material for: The clinical efficacy of herbal medicines containing leeches in the treatment of coronary heart disease: a systematic review and meta-analysis
Source: Front Pharmacol. 2025 Oct 17;16:1643611. doi: 10.3389/fphar.2025.1643611 (PMC12575325; doi:10.3389/fphar.2025.1643611)
Supplement: Supplementary file 2 [file Table2.docx]

**Author(s):**

**Question:** New Comparison compared to placebo for [health problem]

**Setting:**

**Bibliography:** . [Intervention] for [health problem].

| **Certainty assessment** | | | | | | | **№ of patients** | | **Effect** | | **Certainty** | **Importance** |
| --- | --- | --- | --- | --- | --- | --- | --- | --- | --- | --- | --- | --- |
| **№ of studies** | **Study design** | **Risk of bias** | **Inconsistency** | **Indirectness** | **Imprecision** | **Other considerations** | **New Comparison** | **placebo** | **Relative (95% CI)** | **Absolute (95% CI)** |  |  |
| **Traditional Chinese medicine, Symptom, Efficacy（二）** | | | | | | | | | | | | |
| 3 | randomised trials | serious | not serious | not serious | not serious | none | 136/147 (92.5%) | 112/146 (76.7%) | **OR 3.75** (1.81 to 7.73) | **158 more per 1,000** (from 89 more to 195 more) | ⨁⨁⨁◯ Moderate | IMPORTANT |
| **Frequency of angina pectoris** | | | | | | | | | | | | |
| 9 | randomised trials | serious | very serious | not serious | not serious | all plausible residual confounding would reduce the demonstrated effect | 559 | 553 | - | MD **1.14 lower** (1.25 lower to 1.04 lower) | ⨁⨁◯◯ Low | IMPORTANT |

**CI:** confidence interval; **MD:** mean difference; **OR:** odds ratio
