# Supplementary material for: The clinical efficacy of herbal medicines containing leeches in the treatment of coronary heart disease: a systematic review and meta-analysis
Source: Front Pharmacol. 2025 Oct 17;16:1643611. doi: 10.3389/fphar.2025.1643611 (PMC12575325; doi:10.3389/fphar.2025.1643611)
Supplement: Supplementary file 3 [file DataSheet1.pdf]

**Parameter**	**Inclusion Criteria**
Population	Patients with a definite diagnosis of coronary heart disease (diagnostic criteria were in accordance with relevant domestic and foreign guidelines) were enrolled.
Intervention	Chinese herbal medicines containing leech ingredients were used alone or in combination with conventional treatment
Comparator	Conventional treatment, placebo or other Chinese medicine;
Outcome	The improvement rate of angina symptoms, electrocardiogram improvement rate, hemorheology index, incidence of major cardiovascular events and adverse drug reactions were recorded.
Study design	Being an RCT in either parallel or cross-over design
Exclusion criteria	Non-RCT studies, those not clearly mentioning the components of leeches, incomplete data or inability to extract valid data, studies with a follow-up period shorter than 4 weeks or lacking scientific evidence, as well as low-quality studies that have been repeatedly published.
