# Supplementary material for: The clinical efficacy of herbal medicines containing leeches in the treatment of coronary heart disease: a systematic review and meta-analysis
Source: Front Pharmacol. 2025 Oct 17;16:1643611. doi: 10.3389/fphar.2025.1643611 (PMC12575325; doi:10.3389/fphar.2025.1643611)
Supplement: Supplementary file 4 [file Table3.docx]

**Author(s):**

**Question:** New Comparison compared to placebo for [health problem]

**Setting:**

**Bibliography:** . [Intervention] for [health problem].

| **Certainty assessment** | | | | | | | **№ of patients** | | **Effect** | | **Certainty** | **Importance** |
| --- | --- | --- | --- | --- | --- | --- | --- | --- | --- | --- | --- | --- |
| **№ of studies** | **Study design** | **Risk of bias** | **Inconsistency** | **Indirectness** | **Imprecision** | **Other considerations** | **New Comparison** | **placebo** | **Relative (95% CI)** | **Absolute (95% CI)** |  |  |
| **whole blood viscosity** | | | | | | | | | | | | |
| 12 | randomised trials | not serious | serious | serious | serious | all plausible residual confounding would reduce the demonstrated effect | 804 | 642 | - | MD **0.69 lower** (0.73 lower to 0.64 lower) | ⨁⨁◯◯ Low | IMPORTANT |
| **plasma viscosity** | | | | | | | | | | | | |
| 12 | randomised trials | not serious | serious | not serious | serious | none | 804 | 642 | - | MD **0.19 lower** (0.22 lower to 0.17 lower) | ⨁⨁◯◯ Low | IMPORTANT |
| **Fibrinogen** | | | | | | | | | | | | |
| 8 | randomised trials | not serious | serious | not serious | serious | none | 497 | 412 | - | MD **0.63 lower** (0.7 lower to 0.55 lower) | ⨁⨁◯◯ Low | IMPORTANT |

**CI:** confidence interval; **MD:** mean difference; **OR:** odds ratio
